# Intranasal Vaccination Promotes Detrimental Th17-Mediated Immunity against Influenza Infection

**DOI:** 10.1371/journal.ppat.1003875

**Published:** 2014-01-23

**Authors:** Asher Maroof, Yvonne M. Yorgensen, Yufeng Li, Jay T. Evans

**Affiliations:** GlaxoSmithKline Vaccines, Hamilton, Montana, United States of America; National Institutes of Health, United States of America

## Abstract

Influenza disease is a global health issue that causes significant morbidity and mortality through seasonal epidemics. Currently, inactivated influenza virus vaccines given intramuscularly or live attenuated influenza virus vaccines administered intranasally are the only approved options for vaccination against influenza virus in humans. We evaluated the efficacy of a synthetic toll-like receptor 4 agonist CRX-601 as an adjuvant for enhancing vaccine-induced protection against influenza infection. Intranasal administration of CRX-601 adjuvant combined with detergent split-influenza antigen (A/Uruguay/716/2007 (H3N2)) generated strong local and systemic immunity against co-administered influenza antigens while exhibiting high efficacy against two heterotypic influenza challenges. Intranasal vaccination with CRX-601 adjuvanted vaccines promoted antigen-specific IgG and IgA antibody responses and the generation of polyfunctional antigen-specific Th17 cells (CD4^+^IL-17A^+^TNFα^+^). Following challenge with influenza virus, vaccinated mice transiently exhibited increased weight loss and morbidity during early stages of disease but eventually controlled infection. This disease exacerbation following influenza infection in vaccinated mice was dependent on both the route of vaccination and the addition of the adjuvant. Neutralization of IL-17A confirmed a detrimental role for this cytokine during influenza infection. The expansion of vaccine-primed Th17 cells during influenza infection was also accompanied by an augmented lung neutrophilic response, which was partially responsible for mediating the increased morbidity. This discovery is of significance in the field of vaccinology, as it highlights the importance of both route of vaccination and adjuvant selection in vaccine development

## Author Summary

Influenza virus remains a global health risk causing significant morbidity and mortality each year, with the elderly (>65 years) and the very young particularly prone to severe respiratory disease. Scientists are working to develop highly efficacious vaccines capable of eliciting broad cross-clade protection from influenza infection. Adjuvants as well as the route of immunization are known to modulate the type, quality and breadth of immune responses to vaccines. In this study, we demonstrated intranasal vaccination with influenza antigens, and a novel synthetic TLR4-based adjuvant system provided protection against a lethal heterologous viral challenge. Immunization stimulated mucosal influenza-specific IgA antibody responses together with systemic IgG antibodies. While intranasal immunization stimulated the production of protective antibodies, vaccination via this route also promoted the generation of influenza-specific Th17 CD4^+^ T cells. These vaccine-induced Th17 cells increased inflammation and morbidity without contributing to viral clearance following challenge. Antibody neutralization of IL-17A during influenza infection significantly reduced the enhanced lung neutrophilic response, which was partially responsible for mediating the increased morbidity. This discovery is of significance in the field of vaccinology, as it demonstrates the importance of both route of immunization and adjuvant selection in vaccine development.

## Introduction

Influenza infection is globally responsible for up to half a million deaths every year (http://www.who.int/influenza/en/). Through rapid antigenic variation, influenza causes seasonal epidemics and potentially more serious pandemics, which are largely controlled through yearly vaccinations that reduce morbidity and mortality. The vast majority of currently licensed influenza vaccines are based on trivalent inactivated vaccines (TIV). These detergent-split vaccines incorporate purified, inactivated antigens against three viral strains: influenza A (H1N1), influenza A (H3N2), and influenza B strains thereby promoting a humoral immune response towards glycoproteins hemagglutinin (HA) and neuraminidase (NA) [1]. Inactivated TIV vaccines are injected via an intramuscular route and efficacy decreases from approximately 80% in young healthy adults to <60% in the elderly [1]. Efficacy can be even lower during years of antigenic mismatch between vaccines and circulating viruses [2]. The World Health Organization (WHO) estimates that approximately 90% of influenza-associated mortalities in the U.S. occur in the elderly (http://www.who.int/influenza/en/).

Unlike parenteral immunization which stimulates systemic immune responses, mucosal vaccination has been shown to promote both local and systemic immunity [3]. Given that the respiratory mucosa is the initial line of defense against influenza, intranasal immunization offers an attractive route for vaccination against this pathogen. The seasonal intranasal live attenuated influenza vaccine *FluMist* (MedImmune, Gaithersburg, MD, USA) is currently the only intranasal influenza vaccine approved for human use. Combining the current detergent split inactivated vaccine with an effective intranasal adjuvant formulation could greatly improve vaccine efficacy, particularly in the elderly where current vaccines have shown poor efficacy [4].

Monophosphoryl lipid A (MPL) is an acylated diglucosamine derivative of lipid A and is currently the most advanced toll-like receptor 4 (TLR4) adjuvant with an acceptable safety and efficacy profile. MPL adsorbed to alum is incorporated in a recombinant hepatitis B vaccine (*Fendrix*, GlaxoSmithKline Vaccines, Rixensart, Belgium) and a human papillomavirus 16/18 virus-like particle vaccine (*Cervarix*, GlaxoSmithKline Vaccines), both for human use. Further research into structure-function relationships of TLR4-based vaccine adjuvants has led to the development of a new class of synthetic lipid A mimetics, the aminoalkyl glucosaminide 4-phosphates (AGPs). Engineered to specifically trigger TLR4 signaling with varying signaling specificity, potency and safety attributes, some AGPs show promise as vaccine adjuvants and are also capable of eliciting nonspecific protection against a wide range of infectious pathogens [5]–[8].

Using the mouse model of influenza infection, we evaluated the immunogenicity and efficacy of a fully synthetic TLR4 agonist, CRX-601, formulated for mucosal delivery. Intranasal vaccination of mice with a liposomal preparation of CRX-601 combined with inactivated, detergent-split influenza antigens led to 100% survival following heterotypic influenza challenge. In addition to the strong flu-specific IgG and IgA antibody responses, intranasal immunization also promoted the generation of antigen-specific Th17 cells. Vaccinated mice transiently exhibited IL-17A-mediated pathology that presented as increased weight loss and morbidity during early stages of infection. This discovery is of significance in the field of vaccinology, as it highlights the importance of route of vaccination and adjuvant selection when developing vaccination strategies against respiratory pathogens.

## Results

### Intranasal immunization with CRX-601 promotes antigen-specific Th17 cells

**Figure 1:**
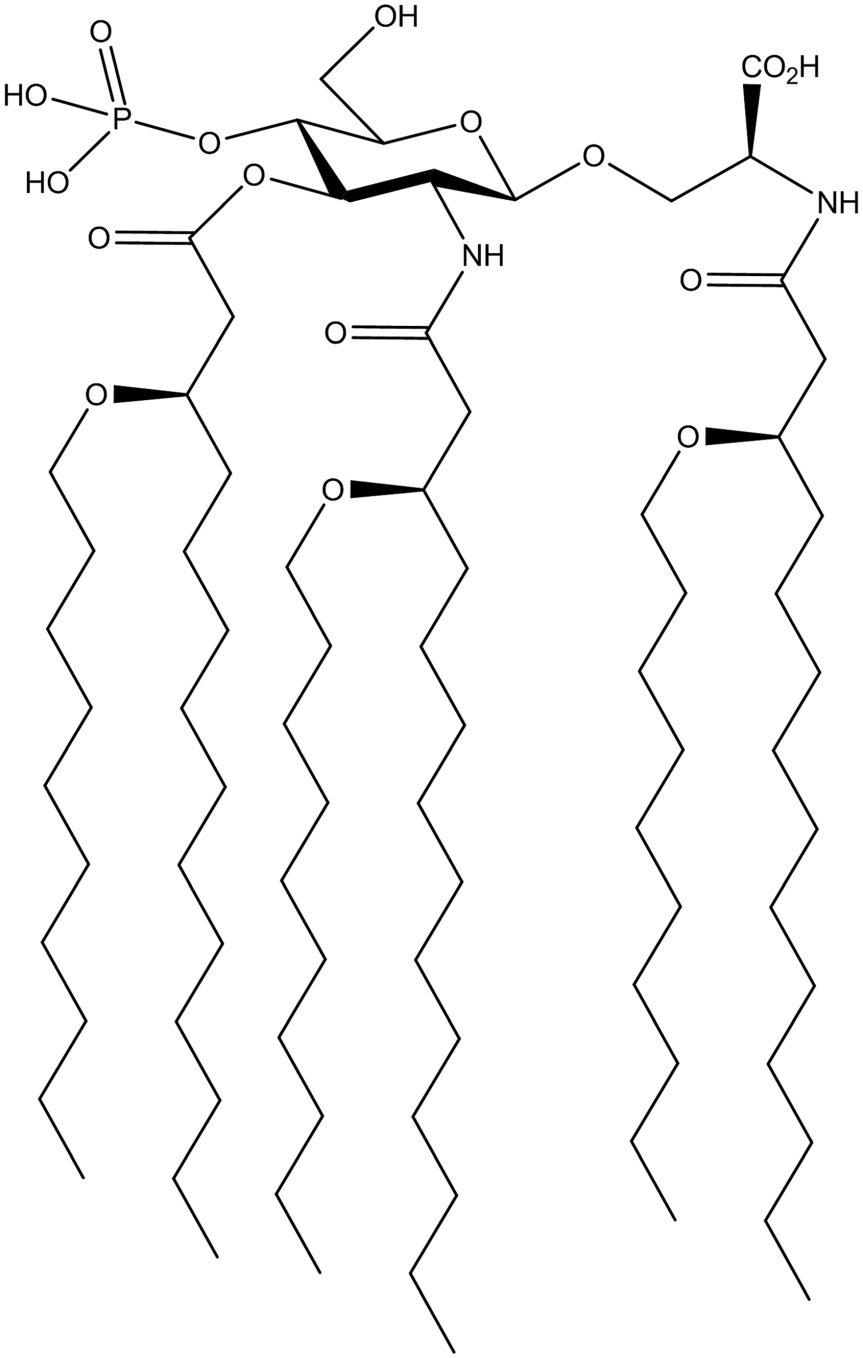
Chemical structure of CRX-601.

**Figure 2:**
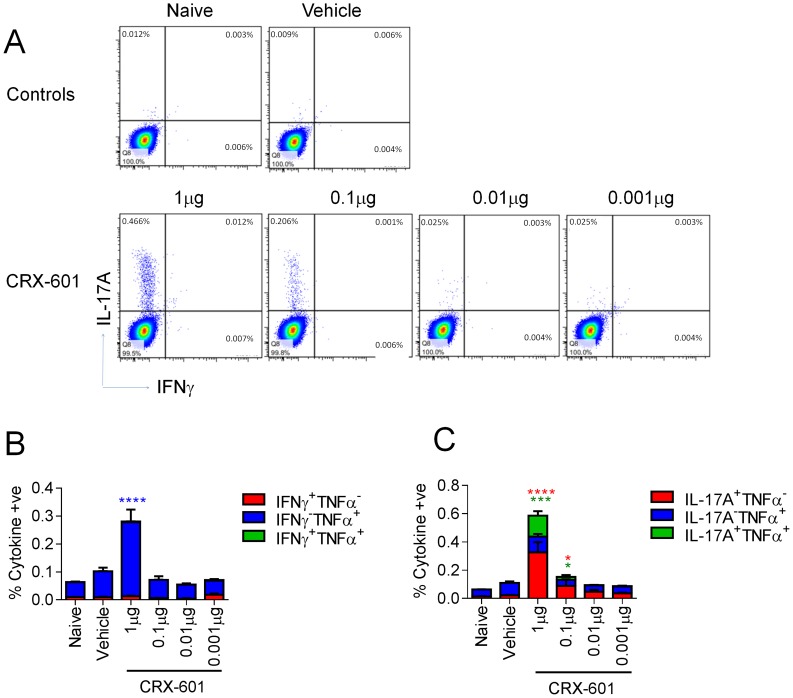
Induction of Th17 cells following intranasal vaccination with liposomal CRX-601 and split flu antigen. Antigen-specific T cell responses in mice vaccinated intranasally with split influenza virus antigen (A/Uruguay/716/2007 (H3N2)) and various concentrations of CRX-601 liposomes were evaluated in the spleen at day 5 post-secondary vaccination. Expression of IL-17A/IFNγ (A), IFNγ/TNFα (B) and IL-17A/TNFα (C) were examined in CD4^+^ T cells. Data shown is representative of 3 independent experiments. Data in B and C are means ± SEM for three replicates analyzed by two-way ANOVA, Bonferroni post test analysis (* p<0.05, ** p<0.01, ***p<0.001, **** p<0.0001 are significantly different from naive controls).

*In vitro* studies in our laboratory have identified important structural requirements for signaling through TLR4 by AGPs [7]–[10]. We have previously described the synthesis of the AGP class of compounds, which are composed of a monosaccharide unit glycosidically linked to an *N*-acylated aminoalkyl aglycon unit [10]. Using an influenza challenge model we investigated the vaccine efficacy of the AGP adjuvant, CRX-601 (Figure 1). The amphipathic nature of CRX-601 readily permits its incorporation into 1,2-dioleoyl-sn-glycero-3-phosphocholine/cholesterol (DOPC/chol) liposomes. The rationale for using liposomes was based on their unique and versatile properties, which offer certain advantages over aqueous dispersions, particularly in the case of mucosal delivery [11], [12]. To generate immunity to influenza antigens, mice were vaccinated via intranasal delivery on days 0 and 21 with increasing concentrations of liposomal CRX-601 combined with detergent split influenza antigens (A/Uruguay/716/2007 (H3N2)). At 5 days post-secondary, we evaluated splenic antigen-specific CD4^+^ T cell responses for helper cell defining cytokines (IL-4, IFNγ and IL-17A) by intracellular cytokine staining (Figure 2A,B). Using the gating strategy in Figure S1A to define CD4+ T cells we did not detect IL-4 (data not shown) or IFNγ (Figure 2A,B), but robust IL-17A production was detected from CD4^+^ T cells of vaccinated mice following antigen-specific restimulation *ex vivo* with split-flu antigen (Figure 2A–C). Frequencies of IL-17A^+^ CD4^+^ T cells were elevated when higher doses of CRX-601 adjuvant were used in the vaccine. A significant proportion of Th17 cells also co-expressed TNFα (∼30% - Figure 2C). Further characterization of vaccine primed CD3^+^CD4^+^IL-17A^+^ cells in the spleen demonstrated that 100% of the IL-17A expressing cells were TCRβ^+^ and TCRγδ^−^ confirming the Th17 phenotype of these antigen specific T-cells. Of note, no cytokine responses were detected in CD8^+^ T cells following antigen-specific restimulation (data not shown). To confirm these findings and demonstrate the extracellular release of IL-17A from CD4^+^ T cells, splenocytes were re-stimulated *in vitro* with split-flu antigen for 72 h and supernatants were quantified for IL-17A. Significantly higher (p<0.05) levels of secreted IL-17A were detected from mice vaccinated with CRX-601 and split-flu antigen as compared to vehicle controls (Figure S1B).

### Vaccination with detergent-split influenza vaccine plus CRX-601 adjuvant induces a strong binding antibody response and protection from influenza virus challenge

**Figure 3:**
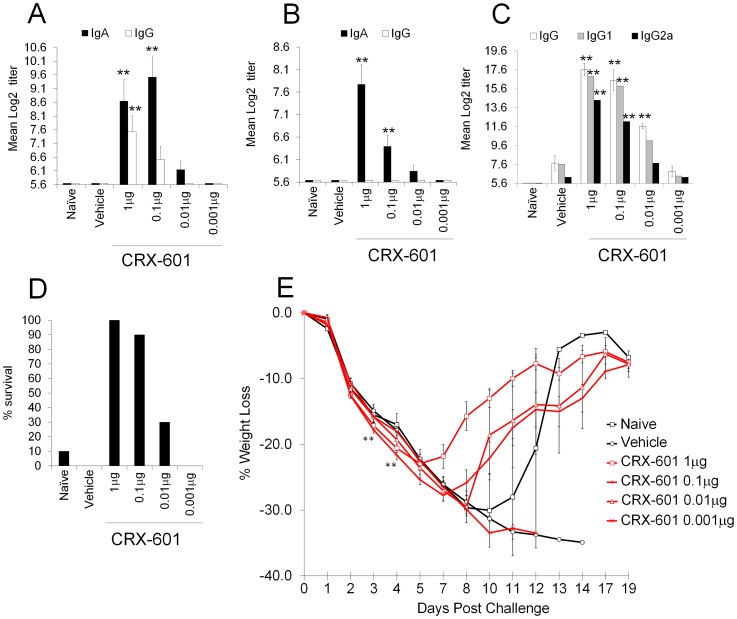
Intranasal vaccination induces both mucosal and systemic immune responses. Anti-influenza total IgG and IgA titers in vaginal (A) and tracheal wash (B) as well as total IgG, IgG1, IgG2a titers in serum samples from mice vaccinated intranasally with split influenza virus antigen (A/Uruguay/716/2007 (H3N2)) and CRX-601 liposomes were examined 14 days post boost vaccination. Percent survival (D) and percentage weight change relative to starting weight (E) following challenge with A/Hong Kong/1968 (H3N2) influenza virus is shown. Data is representative of two independent experiments with 8 mice per group for antibody titers and 10 mice per group for influenza challenge. Data in A, B and C are means ± SEM from 8 mice and E is means ± SEM for 10 mice analyzed by one-way ANOVA, ** p<0.01 denotes significance compared to vehicle control (Dunnet post-test).

A key advantage of mucosal over parenteral vaccination is the induction of both systemic and mucosal antibody responses. We therefore evaluated mucosal antibody titers from tracheal and vaginal wash samples 14 days post-secondary vaccination. Mice receiving adjuvanted vaccines had significantly (p<0.01) higher IgA titers than non-adjuvanted (vehicle) controls in an adjuvant dose-dependent manner (Figure 3A–B). Additionally, influenza-specific IgA could be readily detected at both local (tracheal wash) and distal (vaginal wash) mucosal sites in vaccinated groups with CRX-601 adjuvant included. Serum anti-influenza specific IgG titers from adjuvanted-vaccine groups (1 µg, 0.1 µg and 0.01 µg, p<0.01) were significantly elevated over non-adjuvanted controls (Figure 3C). Anti-influenza serum IgG1 and IgG2a responses were suggestive of a mixed Th1/Th2 profile with a trend towards Th1 in the presence of CRX-601 adjuvant (Figure 3C). To determine vaccine efficacy, mice were challenged with a 5LD_50_ dose of influenza virus (A/Hong Kong/1968 (H3N2)) at day 35 post-secondary vaccination. Compared to control animals, mice vaccinated with higher doses of CRX-601 adjuvant (1 to 0.1 µg), exhibited stronger resistance to influenza challenge with 90–100% survival (Figure 3D) as well as demonstrating a more rapid recovery to starting weight (Figure 3E). Intriguingly, at days 3–4 post challenge, mice vaccinated with higher doses of CRX-601 exhibited significantly (p<0.01) greater weight loss than non-vaccinated control mice. The enhanced weight loss within these groups was transient and the mice began to recover from day 7 onwards (Figure 3E).

The antigen-specific splenic T cell responses of mice that survived the influenza challenge were assessed. The results revealed that vaccine-primed Th17 cells maintain their capacity to secret IL-17A with a fraction also co-expressing IFNγ (Figure S2A–C). In contrast, the influenza infection in non-vaccinated control mice predominantly drove the induction of IFNγ-producing CD4^+^ T cells.

### Induction of antigen-specific Th17 cells by CRX-601 adjuvant is dependent on the route of vaccination

**Figure 4:**
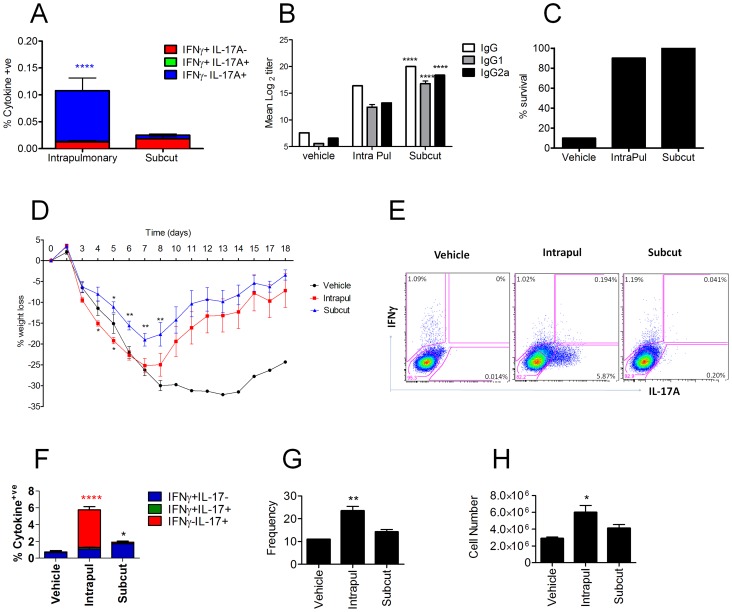
Enhanced vaccine efficacy following subcutaneous immunization. Balb/c mice were vaccinated with CRX-601 liposome (1 µg/mouse) and split influenza virus antigen (A/Uruguay/716/2007 (H3N2)) either via an intranasal (intrapul) or subcutaneous (subcut) route. Splenic T cell responses (A) and serum influenza-specific IgG titers (B) were both examined 5 days post boost vaccination. Percentage survival (C) and percentage weight change (D) following challenge with a lethal dose of A/Hong Kong/1968 (H3N2) influenza virus. Lung influenza specific CD4^+^ T cells responses were evaluated at 5 days post challenge (E, F), in addition to changes in frequency (G) and cell number (H) of Lung Gr-1^hi^ neutrophils. Data from 5 days post boost are means ± SEM for 3 (T-cell) or 8 (Antibody) replicates and is representative of two independent experiments. Data from 5 day post challenge are means ± SEM for three replicates and is representative of two independent experiments. * p<0.05, ** p<0.01, ***p<0.001, **** p<0.0001 denote significance when compared to vehicle treated controls (Figure 4A,F (two-way ANOVA, Bonferroni post test), Figure 4B,C, D,G,H (One-way ANOVA Dunnet post-test)).

Next, we investigated whether generation of the Th17 cells was dependent on the use of CRX-601 adjuvant or the route of vaccination. To address this question, mice were either vaccinated parenterally via the subcutaneous footpad route or via a mucosal intranasal route. Assessment of splenic antigen-specific T cell responses 5 days post-secondary vaccination confirmed Th17 polarization following intranasal administration, while subcutaneous vaccination failed to promote a detectable Th1 or Th17 response (Figure 4A). In contrast, mice vaccinated via the footpad showed a trend towards higher serum antibody responses (8–32 fold) for all isotypes tested compared to mice inoculated via the intranasal route (Figure 4B). Following influenza challenge, both vaccinated groups exhibited similar survival rates irrespective of the route of vaccination (Figure 4C). Importantly, footpad-vaccinated mice did not exhibit the amplified weight loss as noted for the intranasally vaccinated mice (Figure 4D). Analysis of innate and adaptive immune responses in the lung at day 5 post challenge revealed an expansion of vaccine-primed Th17 cells (Figure 4E, F) associated with elevated numbers of neutrophils (Figure 4G, H), specifically in groups of mice vaccinated via the intranasal route. While TCRγδ^+^ cells can be an important source of IL-17 in the lungs the presence and role of these cells in response to influenza infection is not clear. Crowe et al reported the recruitment of IL-17 expressing TCRγδ cells from 2–6 days post infection while Eichelberger et al reported most T-cell recovered prior to day 7 after influenza infection express TCRαβ while TCRγδ cells are found at higher frequency between 7 and 14 days [13], [14]. To determine the TCR phenotype of the CD4^+^IL-17A^+^ cells in the lungs of vaccinated and influenza infect mice lungs from influenza infected mice were evaluated for TCR and IL-17A expression at 4 days post infection. Similar to the results post vaccination 100% of the antigen-specific CD3^+^CD4^+^IL-17A^+^ were TCRβ^+^ and TCRγδ^−^ confirming the Th17 phenotype of the antigen specific cells.

Intranasal influenza vaccination in humans would likely target the upper respiratory tract, whereas the studies described herein used a 20 µL (10 µL/nare) intranasal installation which delivers vaccine components to both the upper and lower respiratory tract (data not shown). It is possible that observed Th17 response and enhanced lung neutrophilia was due to the vaccine components being delivered into the lower airways. To investigate this possibility, mice were treated using low volume (2.5 µL/nare) strict intranasal vaccine administration. Assessment of antigen-specific splenic T-cell responses at 5 days post-secondary vaccination demonstrated an adjuvant dose-dependent Th17 response, albeit lower than previously described for high volume intranasal vaccination (Figure S3A). Serum and mucosal anti-influenza antibody responses were similar to those reported above for intranasal/intrapulmonary vaccination with a CRX-601 adjuvant dose-dependent increase in flu-specific antibody titers (data not shown). To determine if the lower Th17 frequency following strict intranasal vaccination would result in enhanced weight loss and lung neutrophilia following influenza challenge, vaccinated and control mice were challenged with a 5 LD_50_ dose of influenza virus (A/Hong Kong/1968 (H3N2)) at day 35 post-secondary vaccination. Similar to the intranasal/intrapulmonary treatment, mice vaccinated with CRX-601 plus split-flu exhibited significantly (p<0.01) greater weight loss than non-vaccinated controls within 5 days post challenge (Figure S3B). Even with the relatively low precursor frequency prior to challenge, Th17 cells in mice vaccinated with split-flu antigen plus CRX-601were rapidly recruited and expanded to >5% of the CD4^+^ T-cells in the lungs at 5 days post infection (Figure S3C). Likewise, enhanced lung neutrophilia was also noted in groups of mice vaccinated with split-flu plus CRX-601 adjuvant via strict intranasal administration (Figure S3D).

### IL-17A exacerbates influenza-driven morbidity, mediated partially through enhanced lung neutrophilia

**Figure 5:**
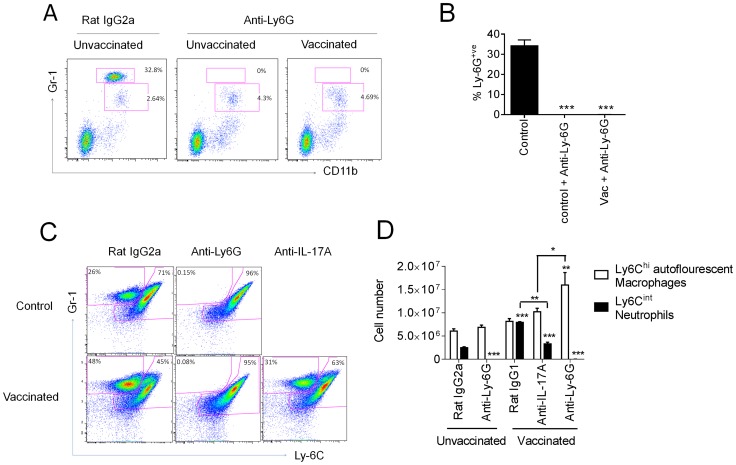
IL-17A neutralization reduces augmented neutrophil response. Mice vaccinated intranasally with split influenza virus antigen (A/Uruguay/716/2007 (H3N2)) and CRX-601 were administered with anti-Ly-6G or anti-IL-17A (100 µg) i.p. one day prior to challenge with influenza virus and then daily for a further 6 days post challenge. Neutrophil depletion efficiency was monitored in peripheral blood 24 h post antibody treatment (A,B). Confirmation of neutrophil depletion (C) as well as enumeration of Gr-1^+^ subsets (D) was examined in the lung 5 days post infection. Data in B and D are means ± SEM for three biological replicates and is representative of two independent experiments. * p<0.05, ** p<0.01, ***p<0.001, are significantly different from control, Rat IgG2a (One-way ANOVA, Dunnet post-test).

**Figure 6:**
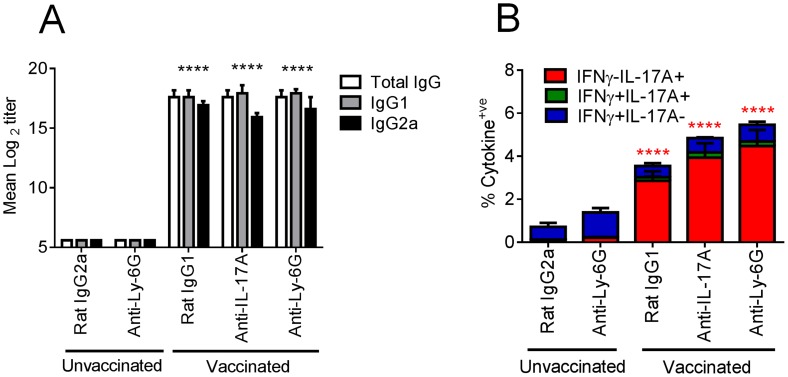
Adaptive immunity is unaffected by IL-17A blockade or neutrophil depletion following viral challenge. Mice vaccinated intranasally with split influenza virus antigen (A/Uruguay/716/2007 (H3N2)) and CRX-601 were administered with anti-Ly-6G (IA8) or anti-IL-17A (100 µg) i.p. one day prior to challenge with influenza and then daily for a further 6 days post challenge. Serum influenza virus specific antibody titers (A) and lung T cell responses (B) were evaluated 5 days post influenza challenge. **** in Figure 6A denotes significance of IgG subtypes compared to unvaccinated IgG2a control using a One-way ANOVA, Dunnet post test. In Figure 6B, **** denotes significance compared to the unvaccinated IgG2a control group using a two-way ANOVA- (Bonferroni- Post-test). Data are means ± SEM for five (A) or three (B) replicates analyzed.

**Figure 7:**
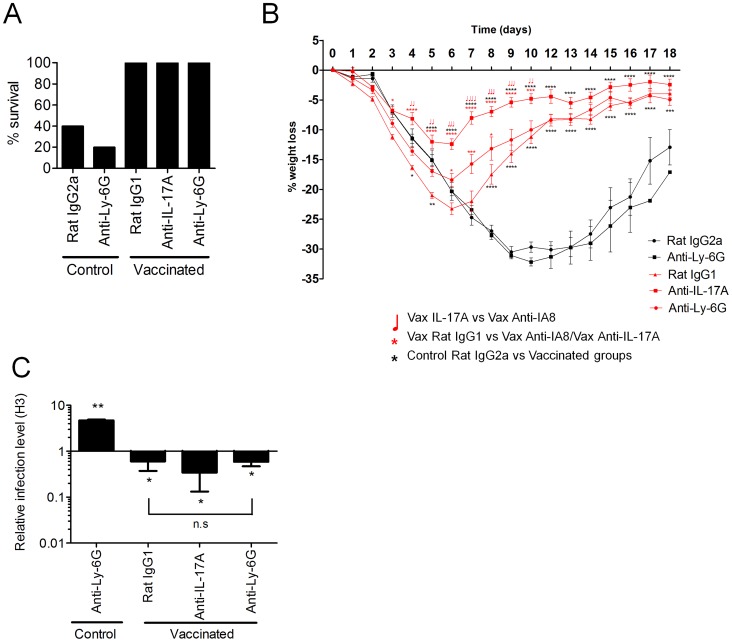
Neutralization of IL-17A and neutrophil depletion ameliorates enhanced weight loss. Mice vaccinated intranasally with split influenza virus antigen (A/Uruguay/716/2007 (H3N2)) and CRX-601 were administered with anti-Ly-6G (IA8) or anti-IL-17A (100 µg) i.p. one day prior to 5LD_50_ challenge with influenza A/Hong Kong/1968 (H3N2) and then daily for a further 6 days post challenge. Control mice were vaccinated with split flu plus vehicle and treated with anti-Ly-6G or isotype control antibodies as indicated. Survival (A) and percentage weight loss (B) were examined following influenza virus challenge (10 mice/group). Black lines indicate weight loss in control (split-flu+vehicle) mice and red lines indicate weight loss in adjuvanted groups treated with the indicated antibody. At day 5 post influenza challenge, (C) viral titers in the lung relative to control mice were evaluated (Rat IgG2a) by RT-PCR. This data is representative of two independent experiments. * p<0.05, ** p<0.01, *** p<0.001, **** p<0.0001 are significantly different from control, Rat IgG2a unless otherwise stated (One-way ANOVA, Dunnet post-test).

Next, we addressed whether the enhanced weight loss observed following influenza virus challenge in intranasally vaccinated mice was due to the expansion of IL-17A-secreting CD4^+^ T cells. Given the neutrophil influx after influenza virus infection in vaccinated mice, we simultaneously explored whether the amplified weight loss was a neutrophil dependent or independent process. To address these questions, mice were depleted of neutrophils by administering the neutrophil-specific anti-Ly6G antibody one day prior to influenza virus challenge. The depletion efficiency was monitored 24 h later by analyzing the frequency of Gr-1^hi^ neutrophils in the peripheral blood. As shown in Figure 5A–B, neutrophil depletion was >99% efficient in both control and vaccinated mice. Within the same experiment, we examined the role of IL-17A by administering a neutralizing anti-IL-17A antibody in additional vaccinated groups of mice. Maintenance of neutrophil depletion and IL-17A neutralization was continued for at least 6 days post challenge through the daily administration of anti-Ly-6G or anti-IL-17A antibody. Analysis of Gr-1 and Ly-6C expression in lung cells 5 days post infection confirmed the loss of neutrophils within this organ in both unvaccinated and vaccinated mice (Figure 5C). A significant (p<0.01) reduction in neutrophil number was also observed in vaccinated mice receiving the neutralizing IL-17A versus vaccinated mice administered with control IgG1 antibody (Figure 5C, D). Quantitation of antigen-specific serum antibody titers in vaccinated groups confirmed a robust response following intranasal immunization (Figure 6A). No significant differences in total IgG, IgG1 and IgG2a levels were measured between any vaccinated groups signifying that the local IL-17A secretion by vaccine primed Th17 cells in the lungs does not appreciably affect antibody titer, at least following influenza challenge. Analysis of antigen-specific T cell responses by intracellular cytokine staining confirmed the priming and expansion of Th17 cells in vaccinated groups, with no significant change in the frequency of Th17 cells following IL-17A blockade or neutrophil depletion (Figure 6B and S4). Vaccinated mice, irrespective of neutrophil depletion or IL-17A blockade, exhibited significant protection against a lethal influenza virus infection compared to unvaccinated controls (Figure 7A). As previously noted, vaccinated mice demonstrated greater weight loss following influenza virus infection compared to unvaccinated mice even following administration of an isotype control antibody. Blocking IL-17A activity with neutralizing antibodies reversed this phenotype, with mice exhibiting less weight loss and quicker recovery to starting weight (Figure 7B). Interestingly, ablating neutrophils in vaccinated mice resulted in an intermediate weight loss phenotype. This indicates that the IL-17A-dependent neutrophil influx during influenza virus infection only partially explains the enhanced weight loss; it is likely that IL-17A is triggering additional responses beyond neutrophil recruitment that influences morbidity. Of note, ablating neutrophils in unvaccinated mice had little effect on the percentage weight loss following influenza virus challenge, which denotes a minor role for neutrophils in the clearance of a primary influenza virus infection.

Lung homogenates including vasculature, tissues and lumen were used to measure lung neutrophil influx we queried if the neutrophils were entering the airway space or residing primarily in the lung vasculature and tissue. To this end we evaluated neutrophil numbers in both bronchoalveolar lavage (BAL) fluids and total lung homogenates from naïve and vaccinated mice at 4 days post influenza challenge. In naïve mice challenged with influenza virus neutrophils (Gr-1^hi^Ly-6C^int^) account for 10.2±1.0% (n = 4) and 14.9±1.4% (n = 4) of the total cells in the lung and BAL, respectively. For intranasal vaccinated (split-flu plus CRX-601) and influenza challenged mice neutrophils accounted for 18.1±3.4% (n = 5) and 37.8±10.1% (n = 5) of the total cells in the lung and BAL, respectively. Vaccinated and challenged mice exhibited a 1.8 and 2.5 fold increases in neutrophils percentage in the lungs and BAL, respectively, as compared to naive/challenged mice demonstrating that neutrophils were trafficking to the lungs and infiltrating the airways at a higher frequency in vaccinated mice.

To determine the impact of IL-17A neutralization or neutrophil depletion on viral replication and clearance, quantitative RT-PCR was used to measure the relative viral load in the lungs of mice at 5 days post challenge. The viral load in all vaccinated groups was significantly less (p<0.05) in comparison to unvaccinated control mice (Figure 7C). In addition, no significant (P>0.05) difference was noted in the viral load between vaccinated groups of mice either depleted of neutrophils or treated with anti-IL-17A antibody. Viral load in unvaccinated mice depleted of neutrophils was significantly higher (p<0.01) than control mice (Rat IgG2a) at 5 days post primary influenza infection (Figure 7C). However, the increased viral load on day 5 did not translate into increased weight loss or reduced survival, suggesting a limited role for neutrophils in resolution of a primary influenza virus infection (Figure 7A–B).

These results confirm that secretion of IL-17A by vaccine-primed CD4^+^ T cells does not contribute to protection against influenza virus in vaccinated mice. These results also highlight that using weight loss as a surrogate marker of viral load and disease protection can in certain circumstances imply false assumptions regarding vaccine efficacy. Collectively, these data suggest that IL-17A exacerbates influenza virus driven morbidity by both neutrophil dependent and neutrophil independent mechanisms.

### Heterogeneous expression of CCR6 on in vivo generated Th17 cells

**Figure 8:**
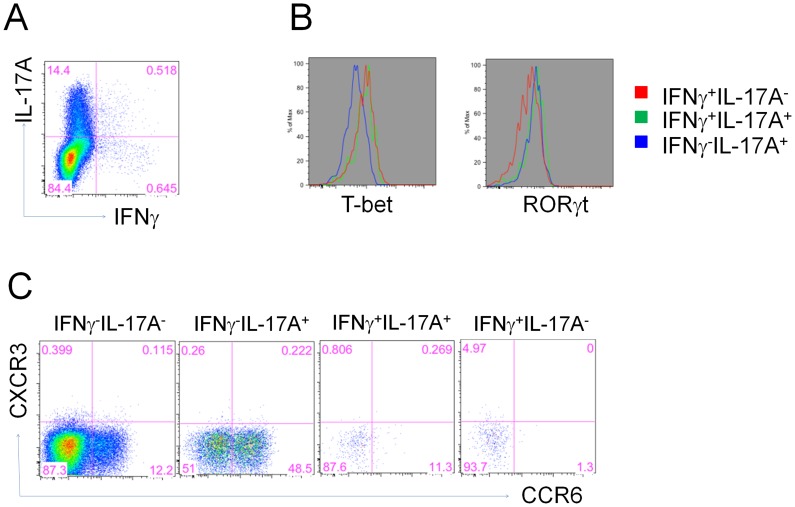
Heterogeneous expression of CCR6 on vaccine primed Th17 cells. 5 days post influenza challenge, lung CD4^+^ T cells from vaccinated mice were examined for antigen-specific IL-17A or IFNγ expression (A), then gated subsets were examined for transcription factors RORγt and T-bet levels (B) as well as CCR6 and CXCR3 expression (C).

Differentiation of Th17 cells has been shown to be controlled by the RORγT transcription factor whereas T-bet has been found to be more selective for Th1 differentiation [15]. To confirm that vaccine-primed IL-17A-secreting CD4^+^ T cells are indeed prototypical Th17 cells, we first assessed the expression of RORγT and T-bet in flu-specific CD4^+^ T cells in the lungs of mice at 5 days following influenza challenge. As expected, IL-17A^+^ CD4^+^ T cells selectively expressed RORγT whereas IFNγ^+^ cells all were positive for T-bet (Figure 8A, B). In contrast, the small fraction of cells secreting both IL-17A and IFNγ expressed both RORγT and T-bet transcription factors. CCR6 chemokine receptor expression is also characteristic of Th17 cells and in combination with CCR4 is regarded as an important homing receptor into certain tissue microenvironments. Examination of CCR6 expression on vaccine-primed Th17 cells demonstrated a heterogenous profile 5 days after influenza challenge with approximately 50% IL-17A^+^ CD4^+^ T cells lacking the chemokine receptor (Figure 8C). Interestingly, dual IL-17A and IFNγ secreting CD4^+^ T cells did not express CCR6.

### Mice primed with a non-lethal dose of influenza virus maintain a Th1 profile after subsequent intranasal vaccination with a CRX-601-adjuvanted influenza vaccine

**Figure 9:**
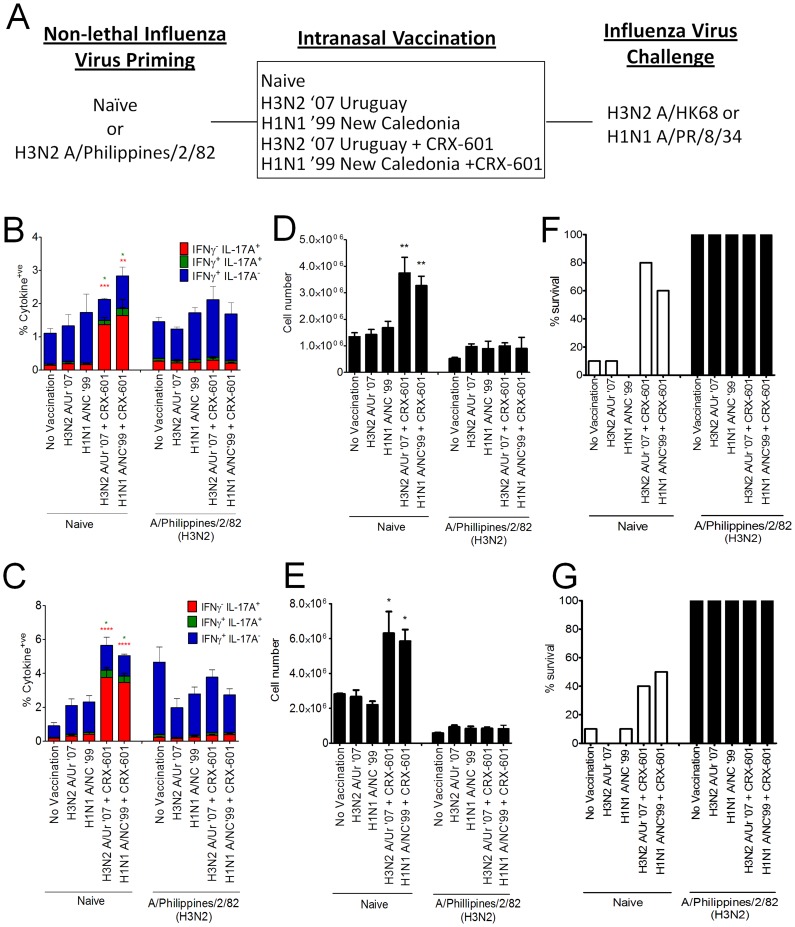
Influenza virus-primed mice maintain a Th1 profile following subsequent intranasal vaccination. Control mice or mice primed with a non-lethal dose of Philippines/2/82/X-79, were subsequently vaccinated intranasally (d28) with split influenza virus antigens derived from either A/Uruguay/716/2007 (H3N2) or A/New Caledonia/20/1999 (H1N1) strains in the presence or absence of CRX-601 liposomes (1 µg/mouse). All groups (10 mice per group) were then challenged with a lethal dose (5LD_50_) of A/Hong Kong/1968 (H3N2) or A/Puerto Rico/8/1934 (H1N1) (A). Antigen-specific lung T cell responses (B,C) and neutrophil numbers (D,E) were evaluated 5 days post influenza challenge with either A/Hong Kong/1968 (H3N2) (B,D) or A/Puerto Rico/8/1934 (H1N1) (C,E). Percent survival for groups challenged with A/Hong Kong/1968 (H3N2) (F) or A/Puerto Rico/8/1934 (H1N1) (G) are also shown. * p<0.05, ** p<0.01, *** p<0.001, **** p<0.0001 are significantly different from no vaccination control (two-way ANOVA, Bonferroni post-test (B,C) and one-way ANOVA, Dunnet post test (D,E).

The majority of adult humans possess a degree of pre-existing immunity to influenza virus. Given that influenza virus infection predominantly generates Th1 mediated immunity, we queried whether mice initially infected with a non-lethal dose of influenza virus would alter their T cell profile following a subsequent intranasal vaccination. To address this question, we initially primed mice with a non-lethal dose of mouse-adapted live influenza (A/Philippines/2/1982/X-79 (H3N2)). Twenty-eight days later, naïve or primed mice were vaccinated intranasally with CRX-601 admixed with either homotypic (A/Uruguay/716/2007 (H3N2)) or heterotypic (A/New Caledonia/20/1999 (H1N1)) detergent split influenza antigens (Figure 9A). Thirty-five days after vaccination, all groups were either challenged with a lethal dose of A/Hong Kong/1968 (H3N2) or A/Puerto Rico/8/1934 (H1N1). Antigen-specific T cell responses were evaluated in the lungs at 5 days post infection.

Intranasal vaccination of mice primed with A/Philippines/2/1982/X-79 (H3N2) exhibited an expanded lung Th1 profile following influenza challenge irrespective of whether homotypic (A/Uruguay/716/2007 (H3N2)) or heterotypic (A/New Caledonia/20/1999 (H1N1)) antigens were used during the intranasal immunization or challenge phase (Figure 9B–C). As expected, non-primed mice vaccinated via the intranasal route without CRX-601 adjuvant failed to generate detectable Th17 responses while CRX-601 adjuvanted mice exhibited a strong Th17 response in the lungs following challenge with H3N2 (Figure 9B) or H1N1 (Figure 9C) influenza strains. Surprisingly, vaccinating mice intranasally with antigen derived from one clade (eg.A/Uruguay/716/2007 (H3N2)) and then challenging mice with influenza from a different strain (A/Puerto Rico/8/1934 (H1N1)) led to the comparable expansion of split-flu specific T cells. This suggests the heterotypic response observed between strains displays a significant degree of cross-reactive T cell recognition. This is surprising considering the completely different clades used in this experiment which encompass over 70 years of antigenic drift and shift between strains. To confirm the vaccine boost response in primed mice, serum was harvested (n = 5) at 21 days post vaccination and evaluated for antibody titers to each of the vaccine and challenge strains. Mice primed with A/Philippines/2/1982/X-79 (H3N2) and vaccinated with A/Uruguay/716/2007 (H3N2) demonstrated a 6 fold boost in anti-A/Uruguay/716/2007 (H3N2) serum IgG titers which were increased another 44 fold with the addition of CRX-601 adjuvant (data not shown). In mice primed with A/Philippines/2/1982/X-79 (H3N2) and vaccinated with A/New Caledonia/20/1999 (H1N1) no boost was detected with antigen alone while the addition of CRX-601 adjuvant resulted in a 3 fold increase in serum antibody titers versus live-virus primed mice (data not shown).

Analysis of the innate immune response at 5 days post challenge confirmed that groups of mice with expanded Th17 cells also had a greater number of neutrophils present in the lungs (Figure 9D–E). Groups of mice initially primed with A/Philippines/2/1982/X-79 (H3N2) generally had lower numbers of lung neutrophils than controls groups, presumably due to the more efficient protection observed in these mice. There was 100% survival in all groups that were initially infected with a non-lethal dose of A/Philippines/2/1982/X-79 (H3N2) (Figure 9F–G) with little clinical sign of infection accompanied by minimal weight loss (data not shown) suggesting that cross-reactive cellular or humoral immunity in the A/Philippines/2/1982/X-79 (H3N2) primed mice was responsible for the high level of protection to heterotypic influenza challenge. In addition, the comparable expansion of split-flu specific CD4 T cells and diminished neutrophil responses suggests that either cross-neutralizing antibodies or cross-reactive CD8 T-cells were responsible for the protection from influenza challenge in primed mice.

## Discussion

The induction of mucosal immunity is an important first line of defense against pathogens such as influenza virus, as the mucosal surface of the respiratory tract is the primary target for viral infection. Coupled with the advantage of needle-free vaccine administration, intranasal immunization additionally promotes both local and systemic immune responses. Pharmaceutical companies are increasingly searching for novel, safe but effective vaccine adjuvants that will facilitate mucosal vaccination. To date, there have been few studies addressing the role of TLR4 adjuvants in vaccine-induced adaptive immunity against influenza, virus particularly following intranasal vaccination, and none have evaluated the existence or role of Th17 cells [5], [16].

In this study, we investigated the efficacy of CRX-601, a synthetic TLR4 agonist, as a vaccine adjuvant to enhance both mucosal and systemic immunity to influenza virus vaccines. When formulated in a liposome, CRX-601 retained potent adjuvant properties which enhanced both cellular and humoral immune responses to co-administered detergent split influenza antigens, providing efficient protection against a homo- and heterotypic influenza virus challenges. However, intranasal vaccination with CRX-601-adjuvanted vaccines led to the induction of a Th17-biased immune response, a phenotype we demonstrate to be dependent on the route of vaccination and was significantly enhanced in the presence of adjuvant. These results are concordant with recent studies demonstrating that intranasal vaccination per se promotes Th17-biased immune responses to a variety of different TLR agonists [17]. The role of IL-17A during influenza virus infection remains controversial, having been reported as both pathogenic and protective [13], [18], [19]. We demonstrate that influenza virus challenge with the drifted A/Hong Kong/1968 (H3N2) strain triggered an antigen-specific expansion of vaccine-primed Th17 cells, which transiently exacerbated inflammation and weight loss after viral infection. Vaccinated mice lost more weight compared to control mice for up to 5 days post infection; however, by 6–7 days post infection, vaccinated mice began to show signs of recovery, ultimately surviving the challenge while naïve or antigen alone-vaccinated mice succumbed to the lethal influenza virus challenge. It is possible that the high dose 5LD_50_ influenza virus challenge contributed to the transient IL-17 induced inflammation and weight loss in vaccinated mice and would not necessarily be reflective of natural exposure to influenza virus. While further studies are needed to explore this vaccine-induced disease exacerbation in larger animal models, a low-dose, non-lethal challenge in vaccinated mice recapitulated the disease exacerbation in mice (data not shown). Of note, antigen-specific Th17 cells were readily detected in the spleen at 5 days boost in adjuvanted vaccine groups but no vaccine-induced weight loss or observable toxicity were noted following the primary or secondary vaccination (data not shown). The administration of neutralizing anti-IL-17A antibodies during viral challenge ameliorated the enhanced weight loss and reduced morbidity in vaccinated mice, verifying a detrimental role for IL-17A in immunity to influenza virus infection. Indeed, IL-17A signaling was also dispensable for viral clearance based on HA (H3) RNA quantitation by real-time RT-PCR. The lack of difference in viral load between vaccinated mice treated with either anti-IL-17A or control antibody was not expected given the dramatic difference in weight loss between both groups following viral infection. This result merely emphasized the fact that IL-17A only serves to enhance inflammation without contributing to protection against influenza virus infection. Of note, although the scope of the study was not to evaluate all sources of IL-17A during infection of vaccinated mice, anti-IL-17A administration would have neutralized IL-17A secreted by all cell types, not just vaccine-primed Th17 cells. Therefore, one can deduce that at least in vaccinated mice infected with influenza virus, IL-17A secretion by either Th17 cells and/or additional sources does not play a role in viral control during the first 5 days of infection.

There is some evidence from other studies that IL-17A can provide a protective antiviral role during primary influenza virus infection without contributing to detrimental inflammatory responses [18], [19]. Primary infection of IL-10 deficient mice leads to greater resistance against a lethal high dose influenza challenge. This enhanced protection was shown to be mediated by an IL-17A-dependent but IFNγ- and perforin-independent process [19]. In partial agreement with our data, Crowe et al demonstrated that in a primary model of influenza infection IL-17RA and IL-17A signaling increased morbidity and was critical for amplified weight loss and inflammation observed between days 2 and 6 post infection [13]. These effects were shown to be mediated by increases in tissue myeloperoxidase due to elevated numbers of lung neutrophils. In their study, the authors demonstrated that γδ T cells are the primary producers of IL-17A during the first week following influenza virus challenge, and the induction of Th17 cells was not detected. Furthermore, using a model of co-infection with influenza A followed subsequently by *Staphylococcus aure*us inoculation, data from the same laboratory indicates a novel mechanism by which influenza A-induced type I IFNs inhibit Th17 immunity and increase susceptibility to secondary bacterial pneumonia [20]. Conjointly, these data suggest that influenza A can suppress the differentiation of Th17 cells but not the production of IL-17A from innate sources such as γδ T cells. In this study, evaluation of TCR expression from antigen-specific IL-17A expressing cells in the lungs or spleens of vaccinated and subsequently influenza infected mice yielded only TCRβ^+^ cells with little or no IL-17A derived from TCRγδ cells in the lungs and spleens. Th17 cell derived IL-22 has also been implicated in homeostasis between pathological airway inflammation and tissue-protective properties following acute injury with bleomycin [21], [22]. Evaluation of lung specific T cell responses by intracellular cytokine staining at 5 days post influenza infection did not reveal any source of IL-22 within a T cell subset or an innate source suggesting IL-22 was not playing a significant role in protection, tissue homeostasis or pathology in this system.

While we can conclusively say that following viral challenge, IL-17A derived from vaccine-induced influenza-specific memory Th17 cells is detrimental in vaccine-induced immunity to influenza based on cytokine neutralization studies, we cannot rule out the IL-23/IL-17 axis playing an important role during the development of an adaptive immune response following vaccination. In a model of tuberculosis infection, both IL-23 and to a certain extent IL-17A, were shown to play a critical role in B cell follicle development [23]. Significantly, vaccination with DNA constructs encoding influenza virus HA and IL-23 cleared more viruses when challenged with influenza virus than HA-constructs alone [24], [25]. These studies support a role for IL-23 in immunity to influenza virus. Transfer of serum from immunized mice to un-vaccinated animals confirmed a role for influenza specific-antibody in mediating protection against viral challenge (data not shown). Although we did not investigate the role of IL-23 at enhancing B cell follicle generation and antibody production following intranasal immunization with CRX-601, there is precedence for the induction of IL-23 by another TLR4 agonist, LPS [26]. B cell recruitment was also substantially decreased in the lungs of influenza virus-infected IL-17-deficient mice [27]. This was associated with reduced CXCR5 expression on B cells and decreased CXCL13 production within the lung tissue of IL-17-KO mice [27].

Several reports also support a role for Th17 cells in triggering an accelerated IFNγ response by CD4^+^ T cells, a process mediated through the induction of the CXCR3 ligand chemokines CXCL9, CXCL10 and CXCL11 [28]. We observed no significant difference, however, in the frequency or number (data not shown) of lung influenza specific Th1 cells 5 days post viral challenge between control and vaccinated mice. Taken together, our data signifies that Th17 cells induced by intranasal vaccination either do not stimulate the necessary chemokines required for enhanced Th1 cell recruitment, or 5 days post challenge is too early to observe any significant differences in CD4^+^IFNγ^+^ T cell recruitment between vaccinated mice and control mice.

Th17 cells are known to have profound effects on neutrophil/monocyte recruitment and activation through the release of IL-6, granulocyte colony-stimulating factor (G-CSF), macrophage inflammatory protein-2 (MIP-2) and keratinocyte-derived chemokine (KC) by tissue epithelial and endothelial cells [29]. Consistent with published reports [30], we also noted an augmented lung neutrophilic response in vaccinated mice infected with influenza. The significant reduction of lung neutrophilia in vaccinated mice treated with anti-IL-17A antibody substantiated the role of vaccine-induced Th17 cells enhancing lung neutrophil recruitment. Independently, this finding does not verify that the enhanced lung neutrophilia was responsible for the increased morbidity and weight loss following influenza infection. To address whether neutrophil recruitment mediated by IL-17A was responsible for the intensified influenza-driven morbidity, we depleted neutrophils in vaccinated mice immediately prior to and during influenza challenge. Based on weight loss, neutrophil ablation of vaccinated mice during influenza infection only partially alleviated symptoms compared to mice treated with anti-IL-17A antibody. Collectively, this suggests that the exacerbated morbidity observed in vaccinated mice is only partially dependent on the enhanced lung neutrophil recruitment and that additional IL-17A-dependent neutrophil-independent mechanisms also contribute to lung pathology. However, neutrophil ablation in vaccinated mice triggered an increase in Gr-1^hi^Ly-6C^hi^ autofluorescent macrophages, a population which normally does not vary in number between unvaccinated and vaccinated groups. We are currently investigating whether depletion of the lung neutrophil compartment artificially creates space for inflammatory phagocytic cell types to be recruited, which would not occur under untreated conditions. Of note, it has previously been shown that neutrophil depletion has a deleterious effect on the clearance of influenza [31]. However, these studies were carried out with an antibody which depletes all Gr-1 expressing cells (RB6-8C5), including monocytes. Using an antibody that specifically depletes neutrophils (1A8), we observed no effect on survival or weight loss in unvaccinated influenza-infected mice; however, a modest increase in viral load was noted. One report suggests that neutrophils magnify and sustain antigen-specific CD8^+^ T-cell responses in the respiratory tract of influenza virus-infected mice [32], although we could not discern any difference in the magnitude or quality of influenza-specific CD8^+^ T cells in this study (data not shown).

The immune response to an adjuvanted influenza virus vaccine via the intranasal route is more difficult to predict in the adult humans due to prior infections with previous influenza strains and/or seasonal immunizations. Herein we demonstrate that primary influenza infection of naïve mice pre-dominantly drives the expansion of influenza-specific Th1 cells, a finding that has been previously reported in both mice [33] and humans [34]. In contrast, intranasal vaccination with CRX-601 and detergent-split influenza antigens promotes the induction of antigen-specific Th17 cells in naïve mice. We further demonstrate that an established influenza-specific Th1 response is preserved subsequent to intranasal immunization with homotypic or even heterotypic split flu antigens. Collectively, this data indicates that intranasal immunization for influenza need not be ruled out, as the most susceptible group, the elderly, are likely to already have a degree of pre-existing Th1-driven immunity to influenza antigens. Likewise, other individuals, including both children and adults who have a pre-existing Th1 immunity to influenza, may also benefit from a CRX-601-adjuvanted intranasal influenza vaccine. In this scenario, intranasal immunization could expand the population of existing Th1 cells specific to influenza, as well as stimulating the appropriate mucosal and systemic antibody response.

The equilibrium between immune protection and tissue inflammation mediated by Th17 cells will define the outcome of vaccine-induced IL-17 responses. In the case of influenza virus, our study draws attention to a need for a more thorough characterization of immune responses following intranasal vaccinations, particularly when novel adjuvants are present in the vaccine formulation.

## Materials and Methods

### Aminoalkyl glucosaminide 4-phosphates

The AGP CRX-601 was synthesized by a highly convergent method as described previously [10]. CRX-601 was purified by flash chromatography on silica gel (to >95% purity) and analyzed as a triethylammonium salt by standard analytical methods. CRX-601 liposomes (CRX-601 DOPC/chol) were prepared by dissolving CRX-601, DOPC and cholesterol in chloroform to a component mass ratio of 0.2∶4∶1 respectively. The chloroform solvent was first evaporated and resulting thin film was re-hydrated with phosphate buffer (pH 6.0). Sonication and high pressure microfluidization was used to reduce the liposomes to the targeted particle size of less than 200 nm. Approximately 99.9% of CRX-601 was incorporated within the liposome as confirmed using the Limulus amebocyte lysate assay, which binds free but not liposome incorporated CRX-601.

### Mice and infections

Female BALB/c mice (6 to 8 weeks of age) were obtained from Charles River Laboratories, Wilmington, MA. For vaccinations, mice anesthetized by i.p administration of ketamine (100 mg/kg) and xylazine (10 mg/kg) were given vaccine by intranasal administration (10 µL/nare), strict intranasal (2.5 µL/nare) or a subcutaneous footpad administration (50 µL). When 10 µL per nare was used for intranasal vaccine administration the vaccine was delivered to both the upper and lower airways (intranasal/intrapulmonary), whereas strict intranasal administration with 2.5 µL per nare was retained in the upper airways (data not shown). All mice were vaccinated on days 0 and 21 with CRX-601 liposomes or liposome vehicle admixed with 0.1 µg HA/mouse using the indicated split influenza antigens. In certain experiments, mice were initially primed with a non-lethal dose (10 µl/nare) of A/Philippines/2/1982/X-79 (H3N2) and subsequently vaccinated intranasally at day 28 post prime with split influenza antigens derived from A/Uruguay/716/2007 (H3N2) or A/New Caledonia/20/1999 (H1N1) (0.1 µg HA/mouse). Mice were challenged intranasally with a 5LD_50_ dose of influenza virus (A/Hong Kong/1968 (H3N2) or A/Puerto Rico/34 (H1N1)) at 35 days following vaccination. Challenged mice were monitored for mortality, clinical signs of disease, and weight change for 20 days following infection. A humane endpoint of 35% (lethal challenge studies) or 15% (non-lethal challenge studies) weight loss was used for all challenge studies. For cell-specific depletion or cytokine neutralization studies, mice were treated with neutrophil-depleting antibody (1A8-Biolegend) or IL-17A-neutralizing antibody (100 µg/mL-Biolegend) administered daily by intraperitoneal injection. All animals were treated in accordance with guidelines established by the U.S. Department of Health and Human Services Office of Laboratory Animal Welfare and the Institutional Animal Care and Use Committee at GSK Vaccines, Hamilton, Montana.

### Serum and mucosal wash collections

Vaginal wash samples were obtained (8 mice per group) from anesthetized mice by washing the vaginal cavity twice with 50 µL of PBS containing 0.01% NaN_3_. Samples were centrifuged to remove cellular debris and the supernatants were collected and stored at −70°C. Anesthetized mice were bled via cardiac puncture and sera collected (8 mice per group) using BD Microtainer Serum Separator Tubes (VWR). Serum samples were stored at −70°C. Anesthetized mice were sacrificed and tracheal wash samples obtained (8 mice per group) by exposing the trachea, inserting a needle through the tracheal wall and flushing with 80 µL of PBS containing 0.01% NaN_3_. Samples were centrifuged to remove debris and the supernatants were collected and stored at −70°C. All tracheal wash samples were visually inspected and discarded if contaminated with blood.

### Tissue preparation

Spleens were isolated at indicated time points and single cell suspensions were prepared by gently pressing through a 100-µM mesh cell strainer (BD Biosciences). Non-perfused lungs were removed, chopped into small pieces and digested with Liberase (300 µg/mL) and DNAse (125 µg/mL) according to the manufacturer's protocol (Roche Diagnostics) followed by passing through a 100-µM mesh cell strainer. For bronchoalveolar lavage collection mice were euthanized by i.p administration of ketamine (100 mg/kg) and xylazine (10 mg/kg) followed by CO2 overdose. The trachea was exposed and cannulated. One milliliter of PBS containing 0.1 mM EDTA and 0.1% BSA was used to flush the airway and collected (∼500 µl final volume). Viable cell counts were determined by Trypan blue exclusion. For intracellular cytokine staining, single cell suspensions (2×10^7^ cells/well) were added to round-bottomed 96-well plates and re-stimulated *in vitro* with 1.5 µg HA/mL of split-flu antigen (A/Uruguay/716/2007 (H3N2)) for 4 h followed by incubation with 10 µg/mL Brefeldin A (Sigma) for an additional 6 h. Following re-stimulation, samples were transferred to V-bottom plates and immediately stained for flow cytometry analysis as described below.

### Flow cytometry

Cells were incubated with 10 ng/mL anti-Fc receptor mAb (BD Biosciences) followed by surface staining with directly conjugated mAbs, including: NC605-conjugated anti-B220(CD45R); FITC-conjugated anti-Gr-1 (RB6-C85), PE-conjugated anti-CD3 (145-2C11), PE-Cy7-conjugated anti-CD8 (53-6.7), allophycocyanin (APC)-conjugated anti-Ly-6C (HK1.4), Alexafluor 700-conjugated anti-CD4 (RM4-5) and anti-Gr-1 (RB6-C85), APC-efluor 780-conjugated anti-CD4(RM4-5) and anti-CD8(53-6.7) (all from eBioscience), PerCP-Cy5.5-conjugated anti-CD3 (145-2C11), BV605-conjugated anti-TCRβ (H57-597) and FITC-conjugated TCRγδ (GL3) (all from BD Biosciences). Minimal background staining was observed using appropriate fluorochrome-labeled isotype controls. Dead cells were stained using Live/Dead Fixable Aqua stain (Invitrogen) as per manufacturer's protocol. Samples were acquired immediately or fixed in 2% paraformaldehyde for subsequent intracellular cytokine staining. Cytokine producing T-helper cells were identified by live cell gate (aqua stain, Invitrogen), forward scatter/side scatter to differentiate lymphocytes followed by a CD3^+^CD4^+^ gate and the resulting population was enumerated for intracellular cytokine staining. Flow cytometric acquisition was performed with a LSR II (BD) and analyzed using FlowJo software (TreeStar). Prior to acquisition, all samples were quantitated by adding polystyrene microbeads (Fluka Analytical) of known amount to each sample. Absolute cell numbers were then enumerated by calculating acquired bead number relative to the number of beads spiked within each sample.

### Intracellular cytokine staining

Fixed cell samples were washed in PBS and stained for intracellular cytokines as per manufacturer's protocol using BD cytofix/cytoperm reagents (BD Biosciences). Cells were then stained with one or more of the following: pacific blue, APC or PECy7-conjugated anti-IFNγ (XMG 1.2) or TNFα (MP6-XT22), FITC-conjugated anti-IL-2 (JES6-5H4), peridinin chlorophyll protein-Cy5.5 or BV421-conjugated anti-IL-17A (eBio17B7, TC11-18H10.1) or anti-IL-10 (JES5-16E3), APC-conjugated anti-IL-12p40 (C17.8) and PE-conjugated anti-IL-22 (1H8PWSR) for 45 min on ice. For transcription factors, T-bet (APC) and RORγt (peridinin chlorophyll protein-Cy5) the permeabilization buffer was supplemented with triton X-100 (0.01%) during the staining phase.

### Specific antibody responses

96-well Maxisorp plates (Nunc) were coated overnight with 0.5 µg/mL of monovalent split flu antigen in PBS. Plates were washed and blocked with Super Block (Skytec) for 1 h at 37°C, then serum or mucosal wash samples were added in diluting buffer (0.01 M TRIS- pH 7.5, 25% FCS, 0.1% Tween 20, 0.2% EDTA, 0.05 µg/mL Thimerosal) and titrated in two-fold serial dilutions across the assay plate. Following 1 h incubation at 37°C, bound antibody was detected with peroxidase goat anti-mouse IgG, IgG1, IgG2a or IgA (1 h, 37°C) and followed by 3,3′,5,5′-tetramethylbenzidine substrate. The optical density was read at 450 nm.

### Luminex

IL-17A production was measured in sample supernatants derived from splenocytes (1×10^5^ cells/well) cultured for 72 h with split-flu antigen in round bottomed 96-well plates. Samples were run using a Milliplex IL-17A Assay Kit (Millipore) and the Luminex 100 System (Luminex Corporation). Assay was performed according to the manufacturer's instructions.

### Real-time RT-PCR

At indicated times, total RNA was isolated from whole lung tissue using an RNeasy kit according to the manufacturer's instructions (Qiagen). RNA was then reverse-transcribed into cDNA using the first-strand cDNA synthesis kit according to the manufacturer's instructions (Invitrogen). Viral titre was quantitated by RT-PCR using Genesig (Primer Design) for human influenza virus subtype (H3) following the manufacturer's instructions. β-actin was used to normalize changes in H3 specific gene expression. PCR samples were run and analyzed on a Biorad myIQ RT-PCR machine.

### Statistics

Statistical analysis was performed using a paired Student *t* test or one way-ANOVA analysis with Bonferrroni, Dunnett or Tukey's multiple comparison post-test. *p*<0.05 was considered significant. All experiments were performed independently at least twice and a minimum of three technical replicates performed for each sample.

## Supporting Information

Figure S1 **Induction of Th17 cells following intranasal vaccination with liposomal CRX-601 and split influenza virus antigen.** Splenic antigen specific T cell responses in mice vaccinated intranasally with split influenza virus antigen (A/Uruguay/716/2007 (H3N2)) and various concentrations of CRX-601 liposomes were evaluated at day 5 post-secondary vaccination. Gating strategy (A) used to examine and quantify intracellular cytokines from CD4^+^ T cells in Figure 2. 10^5^ splenocytes/well were restimulated with 3 concentrations of antigen *in vitro* for 72 h and supernatants were quantified for IL-17A (B). IL-17A data are means ± SEM for 3 mice analyzed. **p<0.01, * p<0.05 are significantly different from naïve controls samples (Two-way ANOVA, Bonferroni post-test).
(TIF)

Figure S2 **Mice recovered from influenza virus challenge maintain Th17 polarization.** Antigen-specific T cell responses in mice that survived influenza virus infection were examined 35 days post viral challenge. Splenic CD4^+^ T cells were restimulated with whole inactivated influenza antigen (A–C) and the expression of IL-17A/IFNγ (A, B) and IFNγ/TNFα (C) was determined by intracellular cytokine staining. Data is representative of 2 independent experiments. Data in B and C are means ± SEM for 3 replicates. * (p<0.05), denotes significance compared to naïve control (two-way ANOVA, Boneferroni post-test).
(TIF)

Figure S3 **Induction of Th17 cells following strict intranasal vaccination with liposomal CRX-601 and split flu antigen.** Balb/c mice were vaccinated with CRX-601 liposome and split influenza virus antigen (A/Uruguay/716/2007 (H3N2)) via strict intranasal (2.5 µl/nare) route. (A) Th17^+^ CD4 T cell responses examined in the spleen at 5 days post boost. Percentage weight change (B) following challenge with lethal dose of A/Hong Kong/1968 (H3N2) influenza virus is shown. Lung influenza specific CD4^+^ T cells responses were evaluated at 5 days post challenge (C) in addition to changes in the total cell number of lung Gr-1^hi^ neutrophils (D). Data are means ± SEM for 3 replicate for cellular responses and 10 replicates for weight loss curves. * p<0.05, ** p<0.01, ***p<0.001, **** p<0.0001 denote significance when compared to vehicle treated controls (A,C (two-way ANOVA, Bonferroni post test), B,D (One-way ANOVA Dunnet post-test).
(TIF)

Figure S4 **Neutralization of IL-17A or neutrolphil ablation does not alter polyfunctional influenza virus-specific T cell responses.** Mice vaccinated intranasally with CRX-601 plus split influenza virus antigen were administered with anti-Ly-6G or anti-IL-17A (100 µg) i.p. one day prior to challenge with influenza virus and then daily for a further 6 days post challenge. Polyfunctional CD4^+^ T cell responses were evaluated in the lung 5 days post influenza virus challenge.
(TIF)

## References

[1] Cox RJ, Brokstad KA, Ogra P (2004) Influenza virus: immunity and vaccination strategies. Comparison of the immune response to inactivated and live, attenuated influenza vaccines. Scand J Immunol 59: 1–15. 14723616 10.1111/j.0300-9475.2004.01382.x

[2] de Jong JC, Beyer WE, Palache AM, Rimmelzwaan GF, Osterhaus AD (2000) Mismatch between the 1997/1998 influenza vaccine and the major epidemic A(H3N2) virus strain as the cause of an inadequate vaccine-induced antibody response to this strain in the elderly. J Med Virol 61: 94–99. 10745239

[3] Pavot V, Rochereau N, Genin C, Verrier B, Paul S (2012) New insights in mucosal vaccine development. Vaccine 30: 142–154. 22085556 10.1016/j.vaccine.2011.11.003

[4] Osterholm MT, Kelley NS, Sommer A, Belongia EA (2012) Efficacy and effectiveness of influenza vaccines: a systematic review and meta-analysis. Lancet Infect Dis 12: 36–44. 22032844 10.1016/S1473-3099(11)70295-X

[5] Baldridge JR, Yorgensen Y, Ward JR, Ulrich JT (2000) Monophosphoryl lipid A enhances mucosal and systemic immunity to vaccine antigens following intranasal administration. Vaccine 18: 2416–2425. 10738099 10.1016/s0264-410x(99)00572-1

[6] Cluff CW, Baldridge JR, Stover AG, Evans JT, Johnson DA, et al (2005) Synthetic toll-like receptor 4 agonists stimulate innate resistance to infectious challenge. Infect Immun 73: 3044–3052. 15845512 10.1128/IAI.73.5.3044-3052.2005 PMC1087352

[7] Stover AG, da Silva CJ, Evans JT, Cluff CW, Elliott MW, et al (2004) Structure-activity relationship of synthetic toll-like receptor 4 agonists. J Biol Chem 279: 4440–4449. 14570885 10.1074/jbc.M310760200

[8] Bowen WS, Minns LA, Johnson DA, Mitchell TC, Hutton MM, et al (2012) Selective TRIF-Dependent Signaling by a Synthetic Toll-Like Receptor 4 Agonist. Sci Signal 5: ra13. 22337809 10.1126/scisignal.2001963 PMC3684200

[9] Baldridge JR, Cluff CW, Evans JT, Lacy MJ, Stephens JR, et al (2002) Immunostimulatory activity of aminoalkyl glucosaminide 4-phosphates (AGPs): induction of protective innate immune responses by RC-524 and RC-529. J Endotoxin Res 8: 453–458. 12697089 10.1179/096805102125001064

[10] Bazin HG, Murray TJ, Bowen WS, Mozaffarian A, Fling SP, et al (2008) The ‘Ethereal’ nature of TLR4 agonism and antagonism in the AGP class of lipid A mimetics. Bioorg Med Chem Lett 18: 5350–5354. 18835160 10.1016/j.bmcl.2008.09.060

[11] Fries LF, Gordon DM, Richards RL, Egan JE, Hollingdale MR, et al (1992) Liposomal malaria vaccine in humans: a safe and potent adjuvant strategy. Proc Natl Acad Sci U S A 89: 358–362. 1729706 10.1073/pnas.89.1.358 PMC48236

[12] Heurtault B, Frisch B, Pons F (2010) Liposomes as delivery systems for nasal vaccination: strategies and outcomes. Expert Opin Drug Deliv 7: 829–844. 20459361 10.1517/17425247.2010.488687

[13] Crowe CR, Chen K, Pociask DA, Alcorn JF, Krivich C, et al (2009) Critical role of IL-17RA in immunopathology of influenza infection. J Immunol 183: 5301–5310. 19783685 10.4049/jimmunol.0900995 PMC3638739

[14] Eichelberger M, Allan W, Carding SR, Bottomly K, Doherty PC (1991) Activation status of the CD4–8− gamma delta-T cells recovered from mice with influenza pneumonia. J Immunol 147: 2069–2074. 1833449

[15] Bettelli E, Oukka M, Kuchroo VK (2007) T(H)-17 cells in the circle of immunity and autoimmunity. Nat Immunol 8: 345–350. 17375096 10.1038/ni0407-345

[16] Baz M, Samant M, Zekki H, Tribout-Jover P, Plante M, et al (2012) Effects of different adjuvants in the context of intramuscular and intranasal routes on humoral and cellular immune responses induced by detergent-split A/H3N2 influenza vaccines in mice. Clin Vaccine Immunol 19: 209–218. 22190392 10.1128/CVI.05441-11 PMC3272927

[17] Zygmunt BM, Rharbaoui F, Groebe L, Guzman CA (2009) Intranasal immunization promotes th17 immune responses. J Immunol 183: 6933–6938. 19890060 10.4049/jimmunol.0901144

[18] Hamada H, Garcia-Hernandez ML, Reome JB, Misra SK, Strutt TM, et al (2009) Tc17, a unique subset of CD8 T cells that can protect against lethal influenza challenge. J Immunol 182: 3469–3481. 19265125 10.4049/jimmunol.0801814 PMC2667713

[19] McKinstry KK, Strutt TM, Buck A, Curtis JD, Dibble JP, et al (2009) IL-10 deficiency unleashes an influenza-specific Th17 response and enhances survival against high-dose challenge. J Immunol 182: 7353–7363. 19494257 10.4049/jimmunol.0900657 PMC2724021

[20] Kudva A, Scheller EV, Robinson KM, Crowe CR, Choi SM, et al (2011) Influenza A inhibits Th17-mediated host defense against bacterial pneumonia in mice. J Immunol 186: 1666–1674. 21178015 10.4049/jimmunol.1002194 PMC4275066

[21] Rutz S, Eidenschenk C, Ouyang W (2013) IL-22, not simply a Th17 cytokine. Immunol Rev 252: 116–132. 23405899 10.1111/imr.12027

[22] Sonnenberg GF, Nair MG, Kirn TJ, Zaph C, Fouser LA, et al (2010) Pathological versus protective functions of IL-22 in airway inflammation are regulated by IL-17A. J Exp Med 207: 1293–1305. 20498020 10.1084/jem.20092054 PMC2882840

[23] Khader SA, Guglani L, Rangel-Moreno J, Gopal R, Junecko BA, et al (2011) IL-23 is required for long-term control of Mycobacterium tuberculosis and B cell follicle formation in the infected lung. J Immunol 187: 5402–5407. 22003199 10.4049/jimmunol.1101377 PMC3208087

[24] Williman J, Lockhart E, Slobbe L, Buchan G, Baird M (2006) The use of Th1 cytokines, IL-12 and IL-23, to modulate the immune response raised to a DNA vaccine delivered by gene gun. Vaccine 24: 4471–4474. 16140432 10.1016/j.vaccine.2005.08.011

[25] Williman J, Young S, Buchan G, Slobbe L, Wilson M, et al (2008) DNA fusion vaccines incorporating IL-23 or RANTES for use in immunization against influenza. Vaccine 26: 5153–5158. 18456374 10.1016/j.vaccine.2008.03.084

[26] Bhan U, Ballinger MN, Zeng X, Newstead MJ, Cornicelli MD, et al (2010) Cooperative interactions between TLR4 and TLR9 regulate interleukin 23 and 17 production in a murine model of gram negative bacterial pneumonia. PLoS One 5: e9896. 20360853 10.1371/journal.pone.0009896 PMC2845620

[27] Wang X, Chan CC, Yang M, Deng J, Poon VK, et al (2011) A critical role of IL-17 in modulating the B-cell response during H5N1 influenza virus infection. Cell Mol Immunol 8: 462–468. 21946434 10.1038/cmi.2011.38 PMC4012931

[28] Khader SA, Bell GK, Pearl JE, Fountain JJ, Rangel-Moreno J, et al (2007) IL-23 and IL-17 in the establishment of protective pulmonary CD4+ T cell responses after vaccination and during Mycobacterium tuberculosis challenge. Nat Immunol 8: 369–377. 17351619 10.1038/ni1449

[29] Fossiez F, Banchereau J, Murray R, Van KC, Garrone P, et al (1998) Interleukin-17. Int Rev Immunol 16: 541–551. 9646176 10.3109/08830189809043008

[30] Bulek K, Liu C, Swaidani S, Wang L, Page RC, et al (2011) The inducible kinase IKKi is required for IL-17-dependent signaling associated with neutrophilia and pulmonary inflammation. Nat Immunol 12: 844–852. 21822257 10.1038/ni.2080 PMC3282992

[31] Fujisawa H (2008) Neutrophils play an essential role in cooperation with antibody in both protection against and recovery from pulmonary infection with influenza virus in mice. J Virol 82: 2772–2783. 18184718 10.1128/JVI.01210-07 PMC2258992

[32] Tate MD, Brooks AG, Reading PC, Mintern JD (2011) Neutrophils sustain effective CD8(+) T-cell responses in the respiratory tract following influenza infection. Immunol Cell Biol 90: 197–205. 21483446 10.1038/icb.2011.26

[33] Roman E, Miller E, Harmsen A, Wiley J, Von Andrian UH, et al (2002) CD4 effector T cell subsets in the response to influenza: heterogeneity, migration, and function. J Exp Med 196: 957–968. 12370257 10.1084/jem.20021052 PMC2194021

[34] Wilkinson TM, Li CK, Chui CS, Huang AK, Perkins M, et al (2012) Preexisting influenza-specific CD4(+) T cells correlate with disease protection against influenza challenge in humans. Nat Med 18: 274–280. 22286307 10.1038/nm.2612

