# Supplementary figures and images for: Intranasal Vaccination Promotes Detrimental Th17-Mediated Immunity against Influenza Infection
Source: PLoS Pathog. 2014 Jan 23;10(1):e1003875. doi: 10.1371/journal.ppat.1003875 (PMC3900655; doi:10.1371/journal.ppat.1003875)

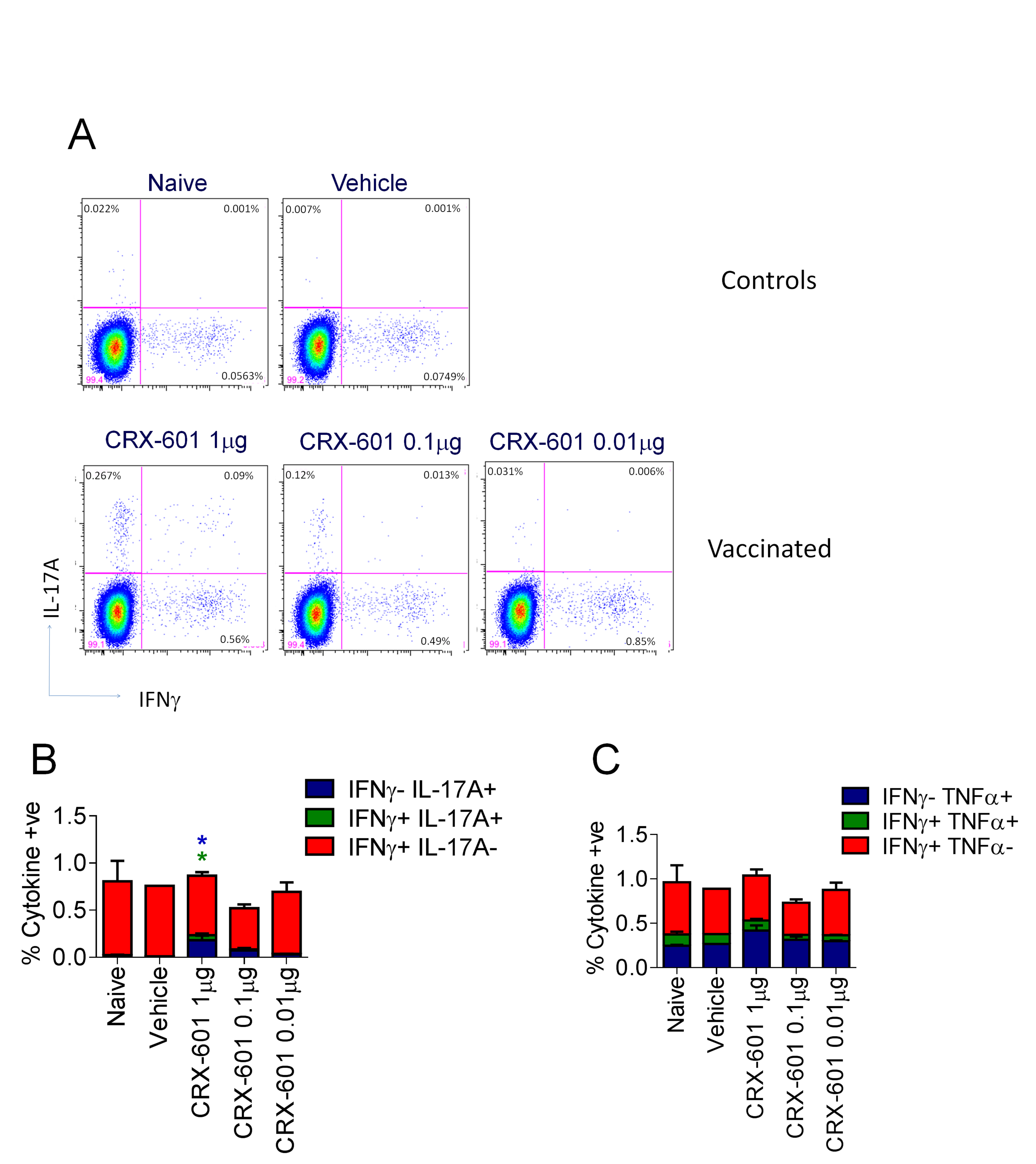

Supplement: Figure S2 — Mice recovered from influenza virus challenge maintain Th17 polarization. Antigen-specific T cell responses in mice that survived influenza virus infection were examined 35 days post viral challenge. Splenic CD4+ T cells were restimulated with whole inactivated influenza antigen (A–C) and the expression of IL-17A/IFNγ (A, B) and IFNγ/TNFα (C) was determined by intracellular cytokine staining. Data is representative of 2 independent experiments. Data in B and C are means ± SEM for 3 replicates. * (p<0.05), denotes significance compared to naïve control (two-way ANOVA, Boneferroni post-test). (TIF) [file ppat.1003875.s002.tif]

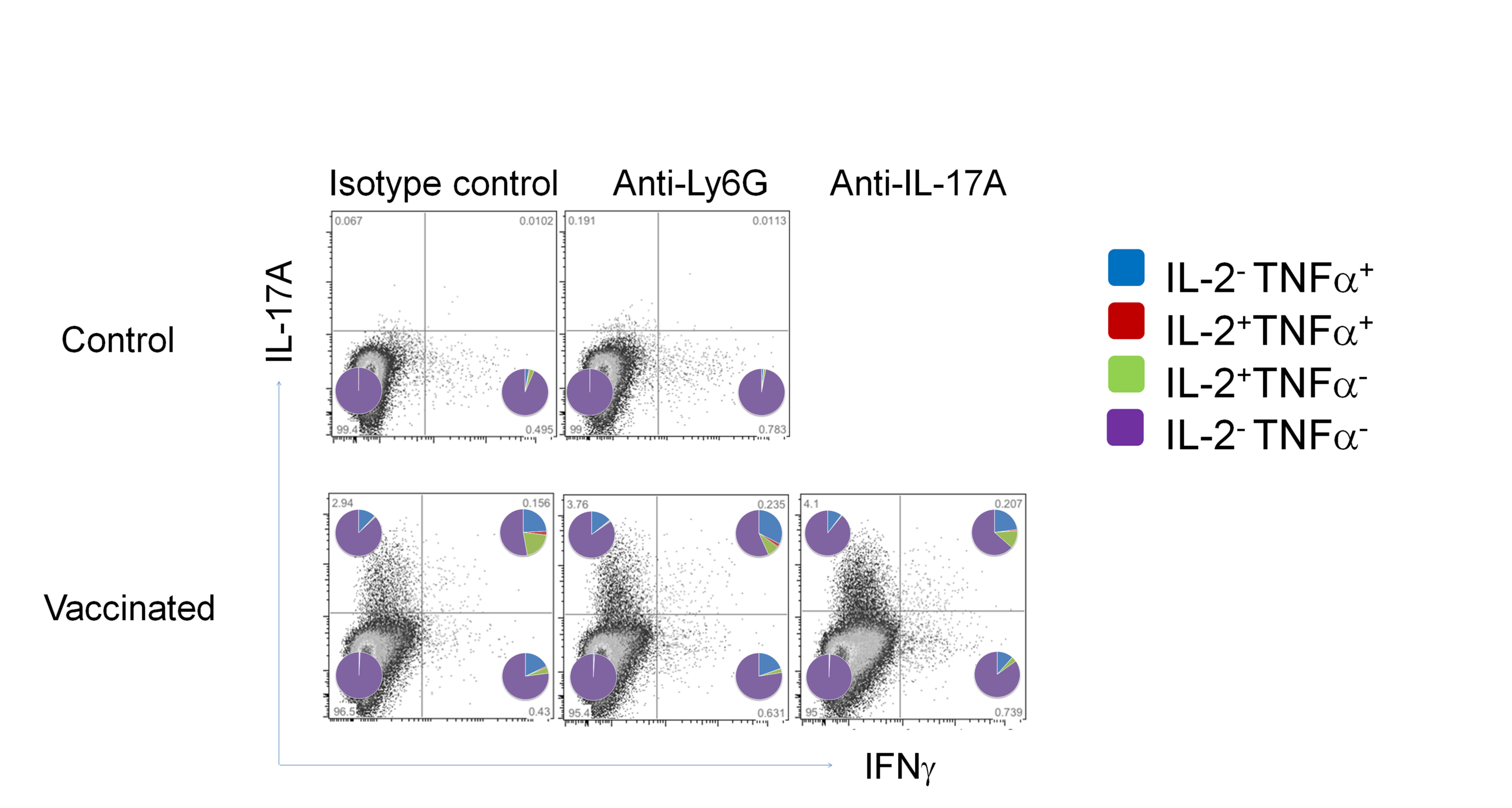

Supplement: Figure S4 — Neutralization of IL-17A or neutrolphil ablation does not alter polyfunctional influenza virus-specific T cell responses. Mice vaccinated intranasally with CRX-601 plus split influenza virus antigen were administered with anti-Ly-6G or anti-IL-17A (100 µg) i.p. one day prior to challenge with influenza virus and then daily for a further 6 days post challenge. Polyfunctional CD4+ T cell responses were evaluated in the lung 5 days post influenza virus challenge. (TIF) [file ppat.1003875.s004.tif]
